# Multiple Therapeutic Mechanisms for a Self-Guided Online Psychoeducation Intervention for Dementia Family Caregivers

**DOI:** 10.1192/j.eurpsy.2026.11583

**Published:** 2026-07-31

**Authors:** S.-T. Cheng

**Affiliations:** Department of Health and Physical Education, The Education University of Hong Kong, Tai Po, Hong Kong

## Abstract

**Introduction:**

Positive Dementia Caregiving in 30 Days (PDC30) is self-guided, fully automated online psychoeducational intervention for dementia caregivers. In a global trial involving 274 caregivers from 43 lower-middle to high income countries, the intervention was found to lower depression and burden, and increase positive gains, compared with 1-month waitlist control. However, the therapeutic mechanism is unclear.

**Objectives:**

To investigate whether self-efficacy beliefs and positive reappraisal coping mediated the intervention effects.

**Methods:**

The inclusion criteria were providing ≥10 hours of care per week, scoring ≥5 on Patient Health Questionnaire-9 (PHQ-9), and passing a simple English comprehension test. Self-efficacy in obtaining respite (SE-OR), self-efficacy in responding to disruptive behaviors (SE-RDB) and self-efficacy in controlling upsetting thoughts (SE-CUT) were measured using the Revised Scale for Caregiving Self-Efficacy. Reappraisal coping was measured using Brief Cope, Positive Reappraisal Subscale (BC-PR). Primary outcome was depression (Patient Health Questionnaire-9 [PHQ-9]) and secondary outcomes were anxiety (brief Profile of Mood States-Anxiety Subscale [POMS-A]), burden (brief Zarit Burden Interview [ZBI]), and positive gains (brief Positive Aspects of Caregiving Scale [PACS]). Following baseline measurement, mediating and outcome variables were re-assessed at 3 weeks and 1 month respectively. 43 individuals having no post-baseline data were excluded, leaving 231 for the mediation analyses. SE-OR and POMS-A were dropped analysis as they were not influenced by the intervention. The general path model PDC30 → mediator (week 3 controlled for baseline) → outcome (1 month controlled for baseline) was estimated using full-information-maximum-likelihood. All mediators and outcomes were tested simultaneously in one integrated model. Indirect effects and confidence intervals were estimated using 5,000 bootstrapped samples. The study was registered with ClinicalTrials.gov, NCT06409455.

**Results:**

The intervention effect on PHQ-9 was mediated by SE-CUT (B = -0.306, 95% CI = -0.624 to -0.106; β = -0.047) and BC-PR (B = -0.477, 95% CI = -0.858 to -0.221; β = -0.074), whereas the effect on burden was mediated by SE-CUT (B = -0.495, 95% CI = -0.965 to -0.192; β = -0.060) and SE-RDB (B = -0.231, 95% CI = -0.574 to -0.034; β = -0.028). On the other hand, the increase in PACS was mediated by BC-PR only (B = 0.402, 95% CI = 0.100 to 0.793; β = 0.053). A full path diagram will be presented at the conference.

**Conclusions:**

The pattern of results suggests that while improved self-efficacy in handling challenging behaviours was partly responsible for PDC30’s effect on caregiver burden, the main drivers of the intervention effects on mood, positive gains, and even burden, were increased use of positive reappraisal and a strengthened belief in one’s ability to avoid negative thoughts.

**Disclosure of Interest:**

None Declared

